# WHAT LINKS DIGITAL SKILLS AND LIFE SATISFACTION? ROLES OF DIGITAL BENEFITS AND LIVING ARRANGEMENTS

**DOI:** 10.1093/geroni/igad104.2214

**Published:** 2023-12-21

**Authors:** Miseon Kang, Seolah Lee, Jungup Lee, Hyo Jung Lee

**Affiliations:** Yonsei University, Seoul, Republic of Korea; Yonsei University, Seoul, Republic of Korea; National University of Singapore, Singapore, Singapore; Yonsei University, Seoul, Republic of Korea

## Abstract

The benefits of equipping enhanced digital skills in late life have been reported on many occasions. Yet, there have been few attempts to understand the association between digital skills and better psychological outcomes for older adults by integrating factors with multiple domains such as social and digital-related factors. This study investigated the causal structure of the association between ‘digital skills’ and ‘life satisfaction’ by specifying a mediator ‘self-reported benefits of digital use’ and a moderated mediator ‘living arrangements’. Using data from ‘2020 Digital Information Gap Survey’ by National Information Society Agency of Korea, the final sample included 1,680 older Koreans aged 55 and over. Analysis was performed by PROCESS macro model 4 and 8 in SPSS. We found a significant positive effect of digital skills on life satisfaction via self-reported benefits of digital use and moderated mediation effect of living arrangements. Specifically, digital skills positively affected self-reported benefits of digital use, and the effect was stronger for older adults living alone. However, we found no moderating effect on the direct path between digital skills and life satisfaction in late life. The conditional direct effect of digital skills on life satisfaction was only significant for older adults living with others. Findings indicate that digital skills can compensate for older adults living alone, who may have fewer sources within a family context. Heterogeneity in family structure needs to be considered when exploring the impact of digital technology use on older adults’ lives.

